# The physiological and psychological effects of cognitive behavior therapy on patients with inflammatory bowel disease before COVID-19: a systematic review

**DOI:** 10.1186/s12876-021-02003-0

**Published:** 2021-12-15

**Authors:** Jie Chen, Xuejie Chen, Yuhao Sun, Ying Xie, Xiaoyan Wang, Ran Li, Therese Hesketh

**Affiliations:** 1 Centre for Global Health, Zhejiang University School of Medicine, 866 Yuhangtang Road, Hangzhou, 310058 People’s Republic of China; 2 Department of Gastroenterology, Central South University, The Third Xiangya Hospital138 Tongzipo Road, Changsha, Hunan 410013 People’s Republic of China; 3 Institute for Global Health, University College London, 30 Guilford St, London, WC1N1EH UK

**Keywords:** Cognitive behavior therapy, Inflammatory bowel disease, Quality of life, Depression, Crohn's disease, Ulcerative colitis

## Abstract

**Objective:**

Cognitive behavioral therapy (CBT) is now included in the treatment of patients with inflammatory bowel disease (IBD) in many settings. However, different clinical trials report different outcomes without consensus. This study aims to evaluate the impact of CBT on the mental state, quality of life and disease activity of patients with IBD.

**Design:**

Systematic review.

**Methods:**

This systematic review searched eligible studies from 1946 to December 8, 2019, in MEDLINE, EMBASE, CINAHL, Cochrane library, ClinicalTrials.gov, PsycINFO, Web of Science for eligible randomized controlled trials (RCT).

**Results:**

Among the initial identified 1807 references, 11 studies met inclusion criteria. CBT was shown to improve patient's quality of life and reduce the level of depression and anxiety post-intervention but was not sustained. Evidence is not enough for the effect of CBT on disease activity, or C-reactive protein level.

**Conclusions:**

CBT has shown short-term positive psychological effects on IBD patients, but there is insufficient evidence for sustained physical and psychological improvements of IBD patients.

*PROSPERO registration*: CRD42019152330.

**Supplementary Information:**

The online version contains supplementary material available at 10.1186/s12876-021-02003-0.

## Strengths and limitations of this study

- The results indicated short-term improvements in depressive symptoms, quality of life measures, after the CBT intervention, though the effect was not sustained.
- Evidence for the efficacy of CBT for disease activity and CRP level in IBD patients is insufficient.
- This study did not investigate the effects of CBT on patients with UC and CD separately, which is consistent with the included studies.

## Introduction

Inflammatory bowel disease (IBD), including Crohn’s disease (CD) and ulcerative colitis (UC), is characterized by chronic inflammation of the gastrointestinal (GI) tract [1]. Recent year have witnessed its increasing high incidence and prevalence, especially in the western world [2]. Both UC and CD cause symptoms like chronic abdominal pain, diarrhea and bloody stools [3–5].

Living with these symptoms has negative impacts on both psychological well-being and health-related life quality. Some studies have found a significant proportion of individuals with IBD suffer from anxiety or depression, including children [6–10]. But the reasons for this association are complex, which may relate to the phenomenon of the Gut–brain axis (GBA), and the way that gut microbes may interact with the central nervous system [11, 12].

The activation of the brain-gut axis includes the release of catecholamines mediated by the autonomic nervous system and stress hormones by the hypothalamus–pituitary–adrenal axis to activate the gastrointestinal response [13–15]. Studies have indicated the relationship between gastrointestinal symptoms and mental state by studying brain–gut interactions in other chronic gastrointestinal diseases [16] and found that this can be a bidirectional relationship [17–19]. Changes in brain–gut interaction are recognized as a component of IBD, the disorders can lead to increased gastrointestinal inflammation and motility, especially under stressful conditions [6, 13, 20–24]. Correspondingly, the deterioration of psychological diseases may also affect gastrointestinal symptoms [10, 17, 18]. Psychosocial factors may be an important correlation factor for IBD pain [25]. In addition, UC and CD may be mitigated using antidepressants [26]. This suggests that psychological problems may influence the natural development of inflammatory bowel disease [27–29].

Among IBD patients, the prevalence of anxiety and depressive symptoms have reached 35% and 22% [30]. With depression or anxiety symptoms, patients with IBD may suffer from weight loss, dietary restriction, et al. resulting in a lower quality of life. For the patients themselves, untreated anxiety and depressive symptoms may lead to unnecessary disabilities and unemployment. For society, it may lead to higher social costs and burdens [31, 32]. Currently, more attention is being paid to the treatments for the psychological problems of IBD patients [11, 12]. A range of psychological therapies is now used as part of the combined clinical treatment in many settings [33, 34]. Such treatments may effectively alleviate symptoms such as anxiety and depression as well as improve physical symptoms in patients with inflammatory bowel disease [35]. A systematic review indicated that cognitive-behavioral therapy (CBT) was the most effective psychotherapy for IBD [36]. It is a psychological therapy helping people to discern and transfer negative patterns affecting emotions and behaviors both at present and in the future. But another systematic review has shown that psychological intervention has no beneficial effect on the relief of IBD in adults while very limited benefits for young people [37]. COVID-19 is reported having associations with mental health and psychiatric illness [38], with may confuse the effect of CBT. Thus, this systematic review chooses population-based studies between 1946 and December 8, 2019, aiming to explore whether it has positive effects on depression, anxiety, stress, disease activity and quality of life in patients with IBD.

## Methods

We designed and report this systematic review according to the Preferred Reporting Items for Systematic Reviews for systematic review protocols (PRISMA-P) 2015 [39]. The International Prospective Register of Systematic Reviews registration number is PROSPERO, CRD42019152330.

### Eligibility criteria

#### Types of studies

##### Inclusion criteria

Randomized controlled trials (RCT) with a follow-up of at least 3 months were included with no language limitation, including studies analysed at different times on the same population.

##### Exclusion criteria

Interventions that were not randomized controlled trials, with incomplete data, or without the full text and secondary studies (e.g. guidelines, reviews) were excluded.

#### Types of participants

##### Inclusion criteria

Studies that referred to population with inflammatory bowel disease diagnosed using any well-established criteria are included.

##### Exclusion criteria

Studies not using standard diagnostic criteria were excluded.

#### Types of interventions

##### Inclusion criteria

Any broadly defined form and type of CBT (such as Acceptance and Commitment Therapy (ACT) and mindfulness therapy, online and offline) were included. The control groups all received IBD treatment as usual (TUA) without any psychotherapy.

##### Exclusion criteria

Studies using non-CBT psychotherapy were excluded.

#### Types of outcome measures

##### Inclusion criteria

Studies using precise and validated screening scales to evaluate psychological indicators where the data provided is complete.

Primary outcomes

Level of depression and anxiety (such as the Hospital Anxiety and Depression Scale (HADS).

Index of disease activity in Crohn's disease and ulcerative colitis (such as Crohn’s Disease Activity Index (CDAI) and Simple Clinical Colitis Activity Index (SSCAI)).

Secondary outcomes

Level of stress.

Levels of Health-Related Quality of Life (HRQoL) (e.g. Inflammatory Bowel Disease Questionnaire (IBDQ).

Level of C-reactive protein (to reflect the severity of inflammation).

##### Exclusion criteria

Studies that did not report the indicators of interest and used non-validated scales are excluded.

### Data search and extraction

#### Search strategy

We searched MEDLINE (PubMed), EMBASE, CINAHL, Cochrane library, ClinicalTrials.gov, PsycINFO, Web of Science and references of all papers for eligible studies from 1946 to December 8 2019. There were no restrictions on language. The full search strategy is in Additional file 1: Appendix S1–S6.

#### Data extraction and management

The title/abstract and full text were screened by two reviewers (XJC and YHS) according to predetermined eligibility criteria and based on pre-prepared extraction sheets (including basic information of the study, participant characteristics (gender, age, type of IBD), intervention (type, frequency, duration) control group (method, duration), follow-up and data for each outcome indicator). The data was verified by the third reviewer (JC).

#### Quality assessment

Quality assessment of the included randomized controlled trials (RCT) was conducted using the Cochrane Collaboration tool [40]. The assessment included selection bias, performance bias, detection bias, attrition bias, reporting bias and others. Each study was checked for its random sequence generation, allocation concealment, blinding of participants and outcome assessment, incomplete outcome data, selective reporting and validated assessment instruments of outcome used.

### Patient and public involvement

No patient involved.

### Ethics approval

This study is exempt from ethics approval because it was conducted based on the published articles.

## Results

### Results of the search

**Fig. 1.**
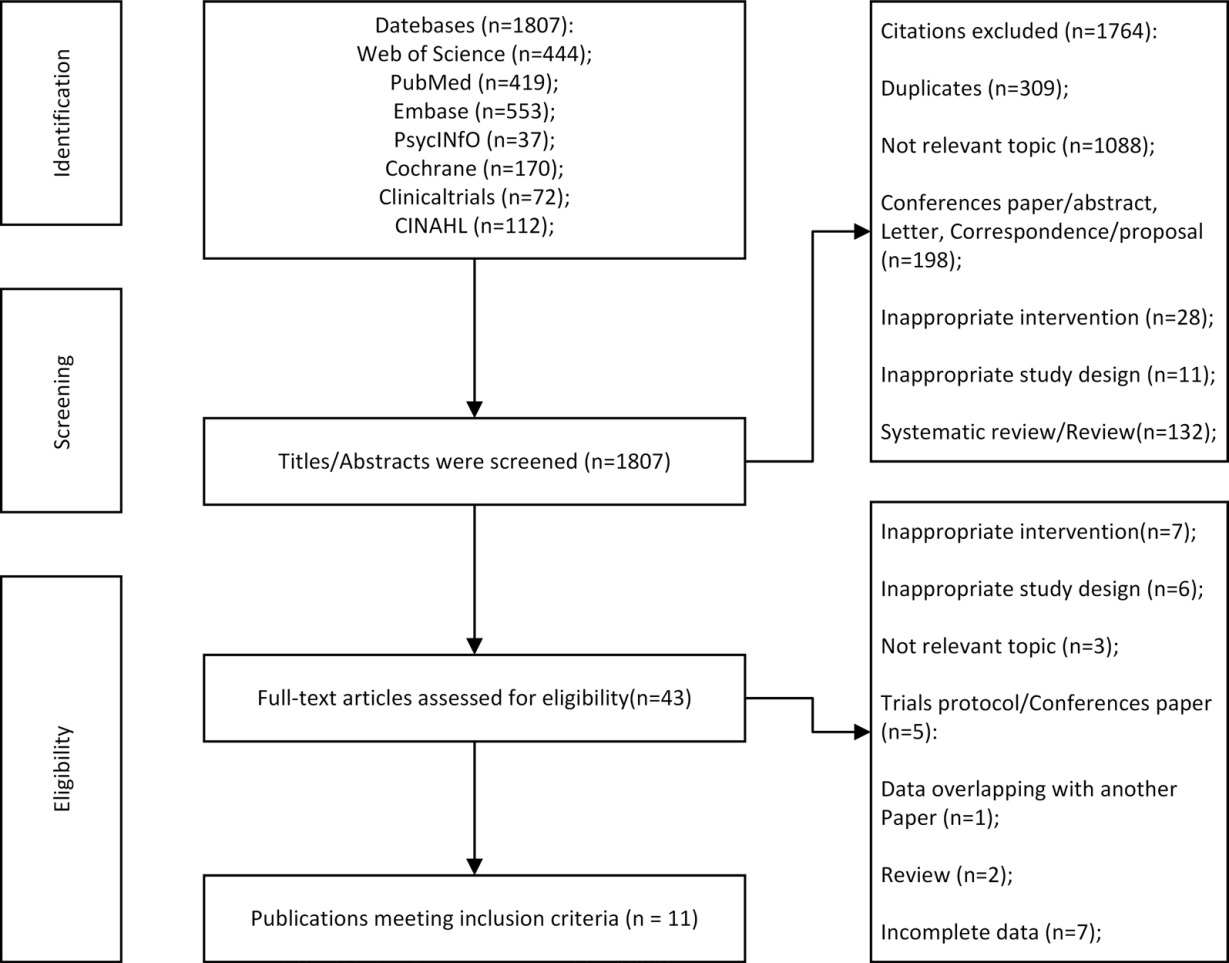
A flow chat diagram of screening and selection processes

Initial screening identified 1807 references (Fig. 1). After filtering by titles/abstracts, 43 studies were left. Eleven studies were found to meet all inclusion criteria based on the full text. Figure 1 shows the exclusion process and the reasons for exclusion of exclusion studies.

### Included studies

#### Types of studies

The 11 included studies were randomized controlled trials investigating the physiological or psychological effects of CBT on IBD patients from 2007 to 2019. Four of these studies are from the United States [41–44], one from Australia with two follow-up times [45, 46], two from the Netherlands [47, 48], one from Ireland [49], one from England [50] and one from New Zealand [51]. The sample size of the studies ranged from 41 to 199, making a total of 995 individuals two studies used the same cohort). Only two studies included only 41 participants [41, 44]. All the included studies reported follow-up data for at least 3 months, with six of them followed for 12 months or more [41, 43, 45–47, 50], and the longest took 2 years [45]. Details can be found in Table 1.

**Table 1:** Characteristics of including studies

References	Region	Design	Duration	Participants (age, sex)	Sample size	Interventions (types, duration)	Control
McCombie et al. [51]	New Zealand	RCT	October 29, 2012, until October 2, 2013	Aged 18–65 years; average age: 38.86; male: 71; female: 128;	Total (n = 199) CD(n = 137) UC (n = 54) IBD-U (n = 8)	8-weeks self-administered CCBT: involved 8 sessions with 62 resources	TAU
Mikocka-Walus et al. [46]	Australia	RCT	21/10/2009 and 14/04/2016	Aged 18 years or over; male: 94; female: 80	Total (n = 174) CD (n = 107) UC (n = 67)	10-weeks CBT (in F2F and CBT group): met weekly at a tertiary hospital for 2 h sessions	SC
Mikocka-Walus et al. [45]	Australia	RCT	21/10/2009 and 14/04/2016	Aged 18 years or over; male: 94; female: 80	Total (n = 174) CD (n = 107) UC (n = 67)	10-weeks CBT (in F2F and CBT group): met weekly at a tertiary hospital for 2 h sessions sessions; 10 W	SC
Wynne et al. [49]	Ireland	RCT	March 2015 and January 2017	Aged between 18 and 65 years; male: 36; female: 43	Total (n = 79) CD (n = 38) UC (n = 41)	8-weeks ACT: consisted of 8 90-min weekly sessions; Each group consisted of 14–16 particles	
Szigethy et al. [44]	America	RCT	September 2002 and August 2007	Aged between 11 and 17 years; male: 20; female: 21	Total (n = 41) CD (n = 29) UC (n = 12)	12-weeks PASCET-PI: consisted of nine modules delivered over 9–11 sixty-minute sessions	TAU
Berrill et al. [50]	England	RCT	January 2011 and May 2013	Aged 18–65 years; average age:44.9; male: 15; female: 51	Total (n = 66) CD (n = 21) UC (n = 45)	16-weeks multi-convergent therapy course plus standard medical therapy (MCT)	Standard medical therapy
Stapersma et al. [9, 48]	Netherlands	RCT	September 2014 and October 1, 2016	Aged 10–25 years; male: 22; female: 48	Total (n = 70) (CD:36; UC: 26; IBD-U:8)	12-weeks PASCET-PI; 10 weekly individual sessions	CAU
Stapersma et al. [47]	Netherlands	RCT	September 2014 and October 1, 2016	Aged 10–25 years; male: 22; female: 48	Total (n = 70) CD (n = 36) UC (n = 26) IBD-U (n = 8)	12-weeks PASCET-PI; 10 weekly individual sessions	CAU
Hunt et al. [42]	America	RCT		average age(M(SD)):35.64 (13.18); male: 48; female: 92	Total (n = 140) CD (n = 67) UC (n = 47) IBD-U (n = 24)	8-weeks self-help CBT: consisted of a self-help book based on skills and principles used in CBT	Psychoeducational workbook
Levy et al. [43]	America	RCT	September 2007 and March 2014	Aged 8 to 17 years; average age:13.5(SD = 2.7); male: 18; female: 167	Total (n = 185) CD (n = 127) UC (n = 58)	18.6-days social learning and cognitive behavioural therapy condition (SLCBT)	ES
Thompson et al. [41]	America	RCT		Aged 11–17; male: 20; female: 21	Total (n = 41) CD (n = 29) UC (n = 12)	PASCET-PI treatment arm participated in 9–11 individual CBT sessions(supplemented by three parent sessions)	TAU

*TAU* treatment as usual, *SC* standard care alone, *CAU* care-as-usual, *ES* educational support condition, *MCT* multiconvergent therapy, *MBSR* mindfulness-based stress reduction, *PASCET-PI* primary and secondary control enhancement therapy-physical illness

#### Populations

Six studies included people over the age of 18 [42, 45, 46, 49–51]. (three of which restricted the maximum age to 65 [49–51]), three studies only targeted people under the age of 18 [41, 43, 44], and two studies included participants aged 10–25 years [47, 48]. In all but two studies [45, 46], the number of female participants greatly exceeded men. All studies included participants with CD and UC, and four of the studies also included a small number of participants from IBD-U [42, 47, 48, 51]. All but two studies [49, 50] had more Crohn's patients than ulcerative colitis. Eight studies excluded patients who had a serious mental illness [41, 44–48, 50, 51], four studies excluded patients with chronic diseases other than IBD [43, 49, 50], and two excluded pregnant women [49, 50].

#### Interventions

All the intervention group in studies used CBT, among which eight studies additionally incorporated the standard [45–50] or usual treatment [41, 51]. Standard treatment represents the current care in clinical practice [47, 48]. And treatment as usual (TAU) refers to therapies maintain the routine naturalistic medical care [41]. The other three only included CBT as targeted measures [42–44], compared with educational, standard, or usual treatment in the control group.

All the included research interventions were off-line CBT except one study that assigned the intervention components to Face to Face (F2F) and online CBT group [51]. Two studies used self-managed CBT therapies for 8 weeks [42, 51]. Hunt et al. also made a self-help book based on skills and principles used in CBT [42] while McCombie et al. provided 8 sessions with 62 resources on a CCBT Web site [51]. It introduces basic knowledge (including diagnosis, diet, treatment et al.) of IBD as well as the introduction and appliance of cognitive restructuring [42]. Four studies used Primary and Secondary Control Enhancement Therapy-Physical Illness (PASCET-PI) [41, 44, 47, 48] within which three lasted 12 weeks [44, 47, 48], while one study did not report the duration [49]. Levy et al. used learning and cognitive behavioral therapy condition (SLCBT) both for parents and children in 18.6 days, encouraging wellness behaviors [43]. Berrill et al. conducted a standardized 16-week 40-min multi-convergent therapy course plus standard medical therapy (MCT) for 16 weeks. The topic contained motivational interview, treatment rationale, mindfulness mediation, theme exploration and relapse prevention [50]. Another study used an 8 90-min weekly sessions ACT provided by a single experienced psychologist [49].

For the control group, all but two studies used standard or usual medical treatment without any psychological treatment for IBD. Three studies used treatment as usual (TAU) [41, 44, 51], and six studies used standard treatment [45–50], For the other two studies, one used an active psychoeducational workbook with the content of the introduction, information and coping ways of IBD for control patients [42], and in the other one, educational support was provided concerning GI system, food labels, and nutrition [43]. Studies have used different measurement scales for each outcome indicator. In the Additional file 1: appendix, all scales used in various studies to report outcomes are shown. (Additional file 1: Table S1).

#### Outcomes

The results of the included studies are listed in Additional file 1: Tables S2 and S3.

### Excluded studies

The exclusion studies and reasons can be found in Additional file 1: Table S4.

### Risk of bias assessment

There is a risk of selection bias. Except for one study [41], other studies used reasonable and correct randomization methods, such as computer-generated random number sequence, web-based randomization protocol and coin flip. The bias of random sequence generation is small. There was no blinding of the personnel in the four studies, and the participants knew their allocation. Participants of two studies were blinded, in three studies bias was unclear. All included studies had varying degrees of attrition (Table 1), but they all reported details of how this was dealt with [42, 51]. All studies used validated assessment instruments to measure the outcome. The quality assessment are listed in Table 2.

**Table 2:** Risk of bias assessment. Methodological quality: review author's judgment about each methodological quality item for each included study

References	Random sequence generation (selection bias)	Allocation concealment (selection bias)	Blinding of participants and personnel (performance bias)	Blinding of outcome assessment (detection bias)	Incomplete outcome data (attrition bias)^†^	Selective reporting (reporting bias)^‡^	Validated assessment instruments of outcome used^§^
McCombie et al. [51]	+	−^a^	−^b^	?^c^	+	?^q^	+
Mikocka-Walus et al. [46]	+	−^d^	−^e^	?	+	+	+
Mikocka-Walus et al. [45]	+	−^d^	+^e^	?	+	+	+
Wynne et al. [49]	+	−^f^	?	?	+	+	+
EVA Szigethy et al. [44]	?	?	?	+	+	+	+
Berrill et al. [50]	+	−^g^	?	?	+	+	+
Stapersma et al. [9, 48]	+	?	?^j^	+^k^	+^l^	+	+
Stapersma et al. [47]	+	?	+^m^	?	+^l^	+	+
Hunt et al. [42]	+	+^n^	?	?	+	?^q^	+
Levy et al. [43]	+	+^o^	?	+^p^	+	+	+
Thompson et al. [41]	?	?	?	?	+	+	+

+Low risk of bias

−High risk of bias

?Unclear risk of bias

^†^The processing methods of attrition data are reported

^‡^The outcome that stated in advance has been reported

^§^Validated assessment tools were used for the assessment

^a^Participants were present when they were randomized

^b^Without blinding; all patients in the TAU group were aware of the CCBT patients receiving CCBT

^c^The author considered that blinding would not have improved the results in favor of the CCBT

^d^Participants are aware they may be offered an intervention or the placebo

^e^Given impossibility of blinding the intervention, we decided to withdraw the information regarding the intervention from the controls. Thus, the controls had been informed they participated in an observational study on mental health in IBD

^f^The author considered that it was not possible to blind participants to their allocated group. To minimize bias, an investigator not involved in recruitment or screening performed randomization, and participants completed all study questionnaires alone

^g^Participants were not blinded as to their allocation following randomization and there was no placebo therapy used in the control group

^h^Participants were not randomly assigned to groups

^i^Participant selection

^j^Only describes the independent curriculum and does not describe the implementation of the blind method in detail

^k^V30% was rated on adherence by at least one rater, and of that 30%, half was evaluated by at least two raters (i.e. 15% of all sessions). Audiotapes were randomly selected to be rated by two of the raters; interrater agreement was globally calculated using Pearson’s correlation between two data columns with (1) all first ratings and (2) all second ratings for all patients and sessions combined

^l^Very low attrition (< 3%)

^m^The interviewer and treating physicians had no access to the files in which the randomization result was described; the youth and their parents not to reveal the trial arm assignment to the interviewer and treating physicians; web-based questionnaires, to be completed at home

^n^Participants were allocated upon receipt of their consent and confirmation of eligibility by a research assistant, who assigned sequential participants based on the results of the coin toss. Thus, allocation was not predetermined and was concealed until assignment

^o^Participants were blind to their group assignment

^p^TThis scale was then mailed back to the study office in a sealed stamped envelope and thus not seen by the therapist

^q^Outcomes reported for completers only

### Results of outcomes

#### Psychological level

##### Depression level

Four studies (339 participants) reported the level of depression of participants immediately after the intervention. Different psychological scales were used: two studies used the Hospital Anxiety and Depression Scale (HADS) [46, 51] and three used the Child Depression Inventory (CDI) [41, 44, 48] two used the Baker Depression Inventory (BDI) [42, 48] and one the Depression Anxiety Stress Scale (DASS) [49]. Stapersma’s study used two scales: BDI and CDI to assess the level of depression.

Five studies (355 participants) reported on the level of depression at follow-up. Two studies claimed that the depression level of participants in the intervention group during follow-up was significantly reduced (*P* = 0.01 [45]; *P* < 0.01 [44]) while no significant improvement was reported in the other studies [41, 46]. Depression scores decreased by 47% in 8 weeks and 45% in 20 weeks compared with baseline (*P* < 0.01) [49]^.^ Mikocka-Walus et al. found that the CBT group has a significant improvement in HADS Depression over 12 months (*P* = 0.02) [46] and Thompson et al. reported that the PASCET-PI group had fewer DSM-IV depressive symptoms compared with TAU (*P* = 0.01) at 3 months, but there was no significance at 9 or 12 months [41].

##### Anxiety level

Three studies (264 participants) reported the level of anxiety of participants post-intervention [42, 48, 49]. Two reported a significant reduction in the level of anxiety after the intervention; one used two scales (Additional file 1: Table S2) (*P* < 0.05 [42]; *P* < 0.01 [48]) Hunt et al. found that those who completed the CBT workbook had a substantial and statistically significant improvement on the STAI at 6-week or 3-month follow up assessment (*P* < 0.05) [42]. Another article also reported the significant effect of time [48]. Four studies (657 participants) reported anxiety levels at follow-up; none reported significant improvement [43, 46, 49, 51]. Though no significance was shown in HADS Anxiety, Mikocka-Walus et al. found a significant improvement in Trait Anxiety over 12 months (*P* = 0.04) [46].

##### Stress level

Two studies (134 participants in total) reported participants' stress levels after the intervention, one reported effectiveness (*P* = 0.04) [49], the other did not (*P* = 0.34) [50]. Four studies (334 participants in total) reported the stress level of participants at follow-up [50, 51], and two reported effectiveness (*P* = 0.04 [34]; *P* < 0.01 [46]). However, Mikocka-Walus's study reported that the stress level of the control group also fell statistically (*P* = 0.01) [46].

##### Quality of life

Three studies (287 participants) reported on the quality of life of participants after the intervention [42, 48, 50]. One study evaluated the quality of life by two scales, IMPACT-III and IBDQ, and it reported the effectiveness of the intervention (IMPACT-III: *P* < 0.01; IBDQ: *P* < 0.05) [48]. Three studies (361 participants) reported on the quality of life of participants at follow-up, CBT group had a greater increase in IBDQ scores over 12 weeks (*P* = 0.01) [51]. Berrill et al. found a higher IBDQ score at 4 months in the trial group than the control (*P* = 0.04) while no significance was shown at 8 and 12 months [50]. However, Mikocka-Walus et al. reported the effectiveness in mental QoL in 12 months in the CBT group (*P* = 0.01) [46].

#### Physiological level

##### Crohn's disease activity

Since the CBT concerned are mainly aimed at psychological symptoms, the studies included few reports on physiological outcomes. These studies reported the degree of Crohn's disease activity of participants after CBT. Wynne’s study reported no effect post-intervention using Crohn's Disease Activity Index (CDAI) [49]. Mikocka-Walus et al. also reported that the CD changed activity was not significant (*P* = 0.67) [46]. On the contrary, Hunt's study reported effectiveness (*P* < 0.01) post-intervention using the Harvey-Bradshaw Index (HBI) [42].

##### Ulcerative colitis disease activity

Only one study reported the weak effect of ACT on disease activity of ulcerative colitis after the intervention (*P* = 0.51) [49]. Two studies (108 participants with UC in total) have reported the invalidity of CBT for follow-up ulcerative colitis disease activity (*P* = 0.51 [49]; *P* = 0.55 [46]).

##### C-reactive protein levels

C-reactive protein (CRP) is an indicator of disease activity or severity of inflammation. The level of CRP will increase during disease activity. Only Wynne et al. reported the CPR level after the intervention (*P* = 0.66) [49], and two studies (194 participants in total) reported the CRP level in the blood of participants at follow-up [46, 49].

## Discussion

This systematic review aimed to determine whether CBT can improve psychological or physical outcomes in IBD patients. Though two previous papers have mentioned the effect of psychological therapy/cognitive-behavioural therapy for IBD, studies published after 2017 have never been covered in any reviews [52, 53]. In our systematic review, we included one study in 2018 and three more in 2019. With the rapid development of CBT in recent years, from treatment contents to delivery methods, self-management has become a new key element inserted in CBT [54]. These new articles focused on self-administration (e.g. a self-help book) in addition to traditional CBT sessions, adding a new dimension to CBT. Besides, we also included CRP as reported outcomes which were ignored by previous systematic reviews. As a biomarker evaluating disease status in patients with IBD in clinical practice, CRP were considered as an important indicator to identify the effectiveness of IBD-related treatment [55].

The results indicated short-term improvements in depressive symptoms and quality of life measures after the CBT intervention, though the effect was not sustained. We also found that CBT does not have significant effects on other indicators such as stress, anxiety, and disease activity. It may relate to the following reasons: (1) when recruiting participants, researchers have not selected according to baseline mental status; participants whose psychological state is relatively normal, are less likely to experience a change in mental health status [56, 57]. (2) the specificity and sensitivity of the detection tools used are not adequate [36]. (3) CBT may not work effectively in reducing stress, anxiety or disease activity for IBD patients.

At the physiological level, we analysed disease activity and CRP level. The results of the systematic review showed that there is insufficient evidence that CBT can improve physical symptoms of IBD patients with whether CD or UC. But other studies have shown that for some other chronic gastrointestinal diseases such as intestinal stress syndrome (ISS), functional dyspepsia and non-cardiac chest pain, and psychotherapy including CBT has been proven to aid in the treatment [36]. The pathogenesis of these gastrointestinal diseases is different from IBD, but sometimes they caused similar symptoms to IBD, which cause these outcomes interesting to be discussed deeper.

The results of Wynne's research are quite different from most of other studies included. Wynne's study specifically designed for the stress response of IBD patients, it focused on increasing mental flexibility related to stress improvement as other studies had done [58, 59]. Participants' disease activity and severity at baseline may have an impact on the efficacy of CBT. Most other studies excluded patients with more severe IBD, and those patients require supplementary surgery or drug treatment while Wynne et al. only included subjects with severe symptoms of mental dysfunction. This may cause other interferences with the results of psychotherapy. Besides, most studies categorized IBD into CD and UC, but have not reported the efficacy of interventions in different types of IBD patients. Given that CD is associated with higher levels of quality of life decline and mood disorders, some research has speculated that CD might be better suited for psychotherapy [37]. The efficacy of CBT in IBD patients with different severity and disease types needs to be analysed in details in future.

A single study used online CBT, and the single positive result reported was that the patient's quality of life could be improved within a 4-week follow-up (IBDQ: *P* = 0.01; SF-12 mental: *P* = 0.03) [51]. A systematic review on whether online CBT has a psychological or physiological effect on patients with gastrointestinal disorders. It shows that after the end of online CBT, severity of symptoms, VS (Visceral Sensitivity Index) and Sheehan Disability Scales have significantly improved in participants with IBS, but no significant results were shown for IBD [60]. These comparisons with the results of the offline CBT research institute may explain that offline CBT intervention may have a more substantial effect than online intervention. However, Mikocka-Walus's study which divided the intervention into two groups, Face to Face (F2F) and online CBT [45, 46]. showed no significant difference between the two groups. But online CBT had higher attrition rates.

Although the included studies inevitably have different attrition rates, they all report methods for managing missing data, which reduces the risk of attrition bias. But the observation that the control group had a higher drop-out rate than the experimental group may skew the results in favor of positive outcomes. In addition, the drop-out rate of CBT is significantly higher than that of other psychotherapy [61]. However, previous studies have shown that the combination of online and offline CBT can effectively reduce the attrition rate and reduce the cost [62, 63], which suggests the need for exploration of other forms of CBT implementation, and efficacy differences and interactions between online and offline CBT in the gastroenterology populations in the future [60]. Also, Wynne's research found that ACT may be an effective measure to relieve psychological stress and depression in IBD patients in the short and long term [49]. Mikocka-Walus found that CBT has a significant role in reducing stress and improving the quality of life of patients [45, 46], future research should further explore this issue.

Existing studies on the use of cognitive behavioral therapy in the treatment of IBD have varied in their interventions and there is no standard criteria or framework. Besides, the scales measuring outcomes like mental states or quality of life are also at an uneven level, making it difficult to evaluate the effect of CBT among IBD patients. Further research is needed for a standardized CBT research and application paradigm.

Limitations of this study are listed as follows: almost no study specifically reported disease activity outcomes classified into CD and UC, therefore, we failed to investigate the effects of CBT on patients with UC and CD respectively and could only discuss the outcome indicators of the total IBD patients. In addition, we didn't set a specific target population, neither concerning the active degree nor the age of the patient. So, we can't get a specific conclusion aiming at the sub-group of IBD patients. However, the purpose of the study was to summarize the results of CBT treatments, as it acts as a foundation for future researches. Thirdly, heterogeneity in terms of different interventions designs and lack of uniform outcomes measures is another limitation. Besides, due to the small number of included studies, we didn’t report publication bias as well.

## Conclusion

Our results show that CBT has short-term improvement effects on depression level and quality of life of patients, but it is not sustained. CBT has no significant impact on physical outcomes. Future studies should pay more attention to the different types of CBT and the long-term effectiveness physiologically and psychologically, for patients with different levels of severity of IBD in different age groups.

## Supplementary Information

**Additional file 1.** Detailed information of included and excluded studies, as well as the searching queries in databases.

## Data Availability

The datasets used and/or analysed during the study available from the corresponding authors on reasonable request.
