# Supplementary material for: The physiological and psychological effects of cognitive behavior therapy on patients with inflammatory bowel disease before COVID-19: a systematic review
Source: BMC Gastroenterol. 2021 Dec 15;21:469. doi: 10.1186/s12876-021-02003-0 (PMC8672154; doi:10.1186/s12876-021-02003-0)
Supplement: Supplementary file 1 — Additional file 1. Detailed information of included and excluded studies, as well as the searching queries in databases. [file 12876_2021_2003_MOESM1_ESM.docx]

**Additional file 1**

Table S1 The outcome measurement scale of included studies

Table S2 Included results of studies

Table S3 Other results of studies

Table S4 Excluded studies

Appendix S1 Searching queries in PubMed

Appendix S2 Searching queries in Embase

Appendix S3 Searching queries in CINAHL with Full Text

Appendix S4 Searching queries in PsycINFO

Appendix S5 Searching queries in Web of Science

Appendix S6 Searching queries in Clinical trials

Additional file 1:Table S1 The outcome measurement scale of included studies

| Outcomes | | McCombie 2016 | Mikocka-Walus 2015,  Mikocka-Walus 2017 | Wynne 2019 | SZIGETHY 2007 | Berrill 2014 | Stapersma 2018, Stapersma 2019 | G. Hunt 2019 | L. Levy 2016 | Thompson 2012 |
| --- | --- | --- | --- | --- | --- | --- | --- | --- | --- | --- |
| Depression | DASS |  |  | √ |  |  |  |  |  |  |
|  | BDI |  |  |  |  |  | √(BDI-II) | √ |  |  |
|  | CDI |  |  |  | √ |  | √ |  |  | √ |
|  | HADS | √ | √ |  |  |  |  |  |  |  |
| Anxiety | DASS |  |  | √ |  |  |  |  |  |  |
|  | STAI |  | √ |  |  |  |  | √ |  |  |
|  | SCARED |  |  |  |  |  | √ |  |  |  |
|  | MASC |  |  |  |  |  |  |  | √ |  |
|  | HADS | √ | √ |  |  |  | √ |  |  |  |
| Stress | DASS |  |  | √ |  |  |  |  |  |  |
|  | PSQ |  |  |  |  | √ |  |  |  |  |
|  | PSS-10 | √ |  |  |  |  |  |  |  |  |
|  | RSRRS |  | √ |  |  |  |  |  |  |  |
| QoL | IBDQ | √ |  |  |  | √ | √ | √(SIBDQ) |  |  |
|  | SF | √(SF-12) | √(SF-36) |  |  |  |  |  |  |  |
|  | IMPACT-III |  |  |  |  |  | √ |  |  |  |
| CD | CDAI |  | √ | √ |  |  |  |  |  |  |
|  | HBI |  |  |  |  |  |  | √ |  |  |
| UC | SSCAI |  | √ |  |  |  |  |  |  |  |
|  | Short Mayo |  |  | √ |  |  |  |  |  |  |

Note: DASS - Depression Anxiety Stress Scale; BDI-II: Beck Depression Inventory—second edition; BDI - Beck Depression Inventory; CDI -Child Depression Inventory; HADS -Hospital Anxiety and Depression Scale; STAI - Spielberger State‐Trait Anxiety Inventory; SCARED - Screen for Child Anxiety Related Emotional Disorders; MASC - Multidimensional Anxiety Scale for Children; PSQ - Perceived Stress Questionnaire; PSS-10 - 0-item version of the Perceived Stress Scale; RSRRS - Revised Social Readjustment Rating Scale; IBDQ - Inflammatory Bowel Disease Questionnaire; SIBDQ - Short Inflammatory Bowel Disease Questionnaire; SF-12 - Social Functioning Questionnaire; SF-36 - Short Form 36 Health Status Questionnaire; CDAI - Crohn’s Disease Activity Index; HBI - Harvey‐Bradshaw Index; SSCAI - Simple Clinical Colitis Activity Index;

Additional file 1: Table S2 Included results of studies

| Study ID | Outcome, measure | | Results for the outcome measure | | | |
| --- | --- | --- | --- | --- | --- | --- |
|  |  |  | Treatment participants (n) | Mean (SD) | Control participants (n) | Mean (SD) |
| Andrew McCombie2016 | anxiety, HADS-A | follow up 12 weeks | 24 | d=-1.07(2.51) | 78 | d=-1.08(2.60) |
|  |  | follow up 24 weeks | 24 | d=-1.63(3.37) | 66 | d=-0.66(2.96) |
|  | stress, PSS-10 | follow up 12 weeks | 24 | d=-1.81(6.21) | 78 | d=-1.49(5.61) |
|  |  | follow up 24 weeks | 24 | d=1.51(7.89) | 66 | d=1.05(7.12) |
|  | depression, HADS | follow up 12 weeks | 24 | d=-1.33(2.24) | 78 | d=-0.86(2.74) |
|  |  | follow up 24 weeks | 24 | d=-0.48(2.68) | 66 | d=-0.51(3.12) |
|  | QoL, IBDQ | follow up 12 weeks | 24 | d=15.39(22.97) | 78 | d=6.63(25.21) |
|  |  | follow up 24 weeks | 24 | d=12.64(26.93) | 66 | d=11.74(29.52) |
| Antonina Mikocka-Walus 2015 | anxiety, HADS | follow up 12 weeks | 51 | 5.9(3.4) | 65 | 5.9 (4.6) |
|  |  | follow up 24 weeks | 42 | 6.5(4.2) | 64 | 6.1 (4.6) |
|  | anxiety, STAI | follow up 12 weeks | 51 | State Anxiety: 34.5 (10.9); Trait Anxiety: 36.1 (11.1) | 65 | State Anxiety: 35.9 (12.8) ; Trait Anxiety: 37.5 (11.8) |
|  |  | follow up 24 weeks | 42 | State Anxiety: 35.9 (13.1); Trait Anxiety: 39.5 (12.5) | 64 | State Anxiety: 35.3 (13.4); Trait Anxiety: 36.9 (13.1) |
|  | stress, RSRRS | follow up 12 weeks | 51 | 474.2(653.7) | 65 | 350.7 (403.4) |
|  |  | follow up 24 weeks | 42 | 301.3(347.5) | 64 | 338.5 (364.2) |
|  | depression, HADS | follow up 12 weeks | 51 | 3.5(2.9) | 65 | 4.1 (4.1) |
|  |  | follow up 24 weeks | 42 | 4.1(3.3) | 6 | 4.5 (4.8) |
|  | QoL, Sf-36 | follow up 12 weeks | 51 | Mental QoL: 48.3(9.2); Physical QoL: 46.7(10.3) | 65 | Mental QoL: 48.1 (11.9); Physical QoL: 47.2(9.8) |
|  |  | follow up 24 weeks | 42 | Mental QoL: 45.5(11.1); Physical QoL: 48.3(10.3) | 64 | Mental QoL: 48.3 (11.5); Physical QoL: 48.3(10.1) |
| Antonina Mikocka-Walus 2017 | anxiety, HADS | follow up 48 weeks | 31 | 5.3 (4.1) | 45 | 5.5 (4.7) |
|  | anxiety, SATI | follow up 48 weeks | 31 | State Anxiety: 32.2 (11.3); Trait Anxiety: 33.6 (10.4) | 45 | State Anxiety: 37.7 (14.7); Trait Anxiety: 38 (14.1) |
|  | stress, RSRRS | follow up 48 weeks | 31 | 5.5 (4.7) | 45 | 442.9 (551.1) |
|  | depression, HADS | follow up 48 weeks | 31 | 3.2 (3.7) | 45 | 3.9 (3.7) |
|  | QoL, Sf-36 | follow up 48 weeks | 31 | Mental QoL: 49.8 (8.8); Physical QoL: 48.8 (10.9) | 45 | Mental QoL: 48.8 (10.9); Physical QoL: 48.8 (8.5) |
| Brona Wynne 2019 | anxiety, DASS-21 | post intervention (8W) | 33 | 5.5(2.0) | 42 | 8.1(2.6) |
|  |  | follow up 20 weeks | 33 | 5.2(1.8) | 42 | 9.1(2.6) |
|  | stress, DASS-21 | post intervention (8W) | 33 | 10.8(2.5) | 42 | 16.9 (2.9) |
|  |  | follow up 20 weeks | 33 | 9.8(2.7) | 42 | 16.3 (2.8) |
|  | depression, DASS-21 | post intervention (8W) | 33 | 5.9(2.3) | 42 | 9.4(2.4) |
|  |  | follow up 20 weeks | 33 | 6.3(2.2) | 42 | 8.9(2.6) |
|  | short mayo | post-intervention (8W) | 33 | 1 (1.05) (95%CI 0 to 1) | 42 | 1 (2.40) (95%CI 0 to 2) |
|  |  | follow up 20 weeks | 33 | 0 (1.05) (95%CI 0 to 1) | 42 | 1 (2.40) (95%CI 0 to 2) |
|  | CDAI | post-intervention (8W) | 33 | 2 (2.10) (95%CI 1 to 3) | 42 | 2 (4.80) (95%CI 0 to 4) |
|  |  | follow up 20 weeks | 33 | 2 (2.10) (95%CI 1 to 3) | 42 | 1 (4.80) (95%CI 0 to 2) |
|  | CRP | post-intervention (8W) | 33 | 2 (3.15) (95%CI 1 to 4) | 42 | 2 (6.00) (95%CI 1 to 6) |
|  |  | follow up 20 weeks | 33 | 1 (4.20) (95%CI 0 to 4) | 42 | 1 (4.80) (95%CI 0 to 4) |
|  | faecal calprotectin | post intervention (8W) | 33 | 55(95%CI 14–164) | 42 | 27(95%CI 14 to 335) |
|  |  | follow up 20 weeks | 33 | 55(95%CI 14–643) | 42 | 28(95%CI 14 to 86) |
| EVA SZIGETHY 2007 | depression, CDI-CP | follow up 12-14 weeks posttreatment | 21 | 10.7(8.0) | 19 | 16.7(11.1) |
| James W. Berrill 2014 | stress, PSQ | follow up 16 weeks | 27 | 0.37(0.14) | 32 | 0.43(0.17) |
|  |  | follow up 32 weeks | 27 | 0.35(0.10) | 32 | 0.41(0.17) |
|  |  | follow up 48 weeks | 27 | 0.35(0.11) | 32 | 0.41(0.17) |
|  | QoL, IBDQ | follow up 16 weeks | 27 | 161(35) | 32 | 145 (39) |
|  |  | follow up 32 weeks | 27 | 155(32) | 32 | 147(38) |
|  |  | follow up 48 weeks | 27 | 150(41) | 32 | 137(38) |
| Luuk Stapersma 2018 | anxiety, HADS-A (for 21–25 years) | post intervention (12W) | 21 | 7.1 (0.7); | 33 | 7.3 (0.8) |
|  | anxiety, SCARED (for 10–20 years) | post intervention (12W) | 14 | 22.9 (2.6) | 33 | 25.0 (2.9) |
|  | depression, CDI | post intervention (12W) | 35 | 7.2 (1.1) | 33 | 7.7 (1.2) |
|  | depression, BDI-II | post intervention (12W) | 35 | 5.9 (1.1) | 33 | 8.2 (1.2) |
|  | QoL, IBDQ | post intervention (12W) | 20 | 179.6 (5.8) | 20 | 171.2 (6.7) |
|  | QoL, IMPACT-III | post intervention (12W) | 50 | 148.1 (2.8) | 50 | 144.9 (3.0) |
| Luuk Stapersma 2010 | anxiety, HADS-A(for 21–25 years) | follow up 24 weeks | 21 | 5.9 (0.6) | 21 | - |
|  |  | follow up 48 weeks | 20 | 6.3(0.6) | 21 | - |
|  | anxiety, SCARED (for 10–20 years) | follow up 24 weeks | 14 | 18.7 (1.9) | 11 | - |
|  |  | follow up 48 weeks | 16 | 18.6 (2.3) | 11 | - |
| Melissa G. Hunt 2019 | anxiety, STAI | post intervention (6W) | 43 | 43 (12) | 45 | 44(13) |
|  | depression, BDI | post intervention (6W) | 43 | 14 (12) | 45 | 14 (11) |
|  | QoL, SIBDQ | post intervention (6W) | 43 | 21 (11.6) | 45 | 22 (12) |
| Rona L Levy 2016 | anxiety, MASC | follow up11 week | 80 | d=-0.2(95% CI -0.9 to 0.5) | 78 | d=-0.2(-0.8,0.5) |
|  |  | follow up 12 weeks | 70 | d=-0.4 (95% CI -1.2 to 0.4) | 67 | d=-0.3(-1.0,0.4) |
|  |  | follow up 24 weeks | 71 | d=-0.6 (95% CI -1.3 to 0.2) | 73 | d=-0.4(-1.2,0.3) |
|  |  | follow up 12 weeks | 67 | d=-0.6 (95% CI -1.5 to 0.3) | 66 | d=-0.8(-1.7,0.1) |
| Thompson, R 2012 | depression, CDI | post intervention (12W) | 21 | 3.03 (9.17) | 19 | 3.89 (15.14) |
|  |  | follow up 36 weeks | 19 | 2.76 (7.64) | 16 | 3.57 (12.74) |
|  |  | follow up 12 weeks | 18 | 2.34 (5.47) | 15 | 3.18 (10.11) |

Additional file 1: Table S3 Other results of studies

| Study ID | Outcome, measure | | Results for the outcome measure | | | |
| --- | --- | --- | --- | --- | --- | --- |
|  |  |  | Treatment participants (n) | Mean (SD) | Control participants (n) | Mean (SD) |
| Andrew McCombie2016 | Social functioning, SFQ | follow up 12 weeks | 24 | d=-0.18(2.60) | 78 | d=-0.86(2.23) |
|  |  | follow up 24 weeks | 24 | d=-0.03(2.71) | 66 | d=-0.76(2.87) |
|  | coping strategies, Brief COPE | follow up 12 weeks | 24 | adaptive problem-focused: d=-0.64(4.83); adaptive emotion-focused: d=-0.05(5.58); maladaptive: d=-1.17(2.96) | 78 | adaptive problem-focused: d=-1.01(4.31); adaptive emotion-focused: d-1.49(4.31); maladaptive: d=-1.99(4.14) |
|  |  | follow up 24 weeks | 24 | adaptive problem-focused: d=-2.52(3.31); adaptive emotion-focused: d=-1.32(4.32); maladaptive: d=-0.36(3.86) | 66 | adaptive problem-focused: d=-2.07(4.41), adaptive emotion-focused: d=-3.60(4.54), maladaptive: d=-2.00(3.67) |
| Antonina Mikocka-Walus 2015 | adaptive and maladaptive coping styles, COPE | follow up 12 weeks | 51 | Adaptive coping: 42.1 (13.6); Maladaptive coping: 9.8 (2.6) | 65 | Adaptive coping: 37.1 (11.2); Maladaptive coping: 10.5 (3.3) |
|  |  | follow up 24 weeks | 42 | Adaptive coping: 40.5 (12.9); Maladaptive coping: 10.6 (3.7) | 64 | Adaptive coping: 37.5 (11.7); Maladaptive coping: 10.5 (3.6) |
|  | IBDSCCQ, TTTM | follow up 12 weeks | 51 | Pre-contemplation: .3 (1.9); Contemplation: 6.6 (1.9); Preparation: 6.5 (2.4); Action: 6.3 (2.3); Maintenance: 7.7 (1.7) | 65 | Pre-contemplation: 4.2 (1.9); Contemplation: 6.2 (2.1); Preparation: 6.1 (2.2); Action: 5.8 (2.3); Maintenance: =7.1 (1.9) |
|  |  | follow up 24 weeks | 42 | Pre-contemplation: =4.2 (1.9); Contemplation: 6.6 (1.6); Preparation: 6.4 (2.3); Action: 6.1 (2.3); Maintenance: 7.3 (1.8) | 64 | Pre-contemplation: M0=4.4 (1.6); M6=4.2 (1.9) ; M12=4.7 (1.9)  Contemplation: 6.4 (1.9); Preparation: 6.1 (2.1); Action: 5.9 (2.3); Maintenance: 7.3 (1.9) |
|  | HB | follow up 12 weeks | 57 | 136.4 (14.8) | 62 | 142.3 (17.2) |
|  |  | follow up 24 weeks | 54 | 136.6 (13.9) | 61 | 141.3 (17.1) |
|  | Platelet | follow up 12 weeks | 57 | 260.8 (64.7) | 62 | 272.1 (74.8) |
|  |  | follow up 24 weeks | 54 | 267.1 (67.1) | 61 | 263.5 (76.3) |
|  | WCC | follow up 12 weeks | 57 | 8.3 (14.8) | 62 | 6.9 (2.5) |
|  |  | follow up 24 weeks | 54 | 6.1 (1.9) | 61 | 6.9 (2.5) |
| Antonina Mikocka-Walus 2017 | adaptive and maladaptive coping styles, COPE | follow up 48 weeks | 31 | Adaptive coping: 43.5 (17.5); Maladaptive coping: 11.6 (10.3) | 45 | Adaptive coping: 41.1 (24.1); Maladaptive coping: 12.2 (9.9) |
|  |  | follow up 48 weeks | 31 | Pre-contemplation: 4.6 (2.2); Contemplation: 6.5 (2.1); Preparation: 6.8 (2.1); Action: 4=6.4 (2.1); Maintenance: 7.5 (1.6) | 45 | Pre-contemplation: 4.9 (2.9); Contemplation: 6.1 (2.7); Preparation: 6.7 (3.1); Action: 6.3 (3.3); Maintenance: 7.6 (2.4) |
|  | HB | follow up 48 weeks | 33 | 140.9 (17.1) | 42 | 136.4 (14.7) |
|  | Platelet | follow up 48 weeks | 33 | 258.9 (63.4) | 42 | 269.8 (77.1) |
|  | WCC | follow up 48 weeks | 33 | 6.6 (2.4) | 4 | 6.4 (1.7) |
| EVA SZIGETHY 2007 | Psychiatric Diagnoses, K-SADS-PL | follow up 12-14 weeks posttreatment | 21 | 1.0(1.2) | 19 | 2.4(2.3) |
|  | Cognitive Processing, PCSC | follow up 12-14 weeks posttreatment | 20 | 63.3(6.5)* | 18 | 54.7(14.6)* |
|  | Global Functioning, CGAS | follow up 12-14 weeks posttreatment | 21 | 69.9(6.7)* | 19 | 69.9(6.7)* |
| Melissa G. Hunt 2019 | Gastrointestinal symptom-specific anxiety and hypervigilance, VSI | post-intervention (6W) | 43 | 35 (19) | 45 | 36 (16) |
|  | gastrointestinal-specific cognitions GI-COG | popost-intervention6W) | 43 | 22 (15) | 45 | 26 (15) |
| Rona L Levy 2016 | Parental response to pain behaviour arent report only), ARCS | follow up 1 week | - | d=-0.4 (95% CI -0.6 to -0.3) | - | - |
|  |  | follow up 12 weeks | - | d=-0.4(95% CI -0.5 to -0.2) | - | - |
|  |  | follow up 24 weeks | - | d=-0.4 (95% CI -0.5 to -0.3) | - | - |
|  |  | follow up 48 weeks | - | d=-0.4 (-0.6 to -0.3) | - | - |
|  | Child catastrophizing and pain-coping skills (Parent and Child Report), PRI | follow up 1 week | - | distract/ignore: d=0.3(95% CI 0.1 to 0.5); Catastrophizing: d=0.1(95% CI -0.3 to 0.0) | - | distract/ignore: d=-0.0(95% CI -0.2 to 0.2); Catastrophizing: d=-0.0(95% CI -0.2 to 0.1) |
|  |  | follow up 12 weeks | - | distract/ignore: d=0.3(95% CI 0.1 to 0.5); Catastrophizing: d=-0.2(95% CI -0.3 to 0.0) | - | distract/ignore: d=0.0(95% CI -0.2 to 0.3); Catastrophizing: d=0.1(95% CI -0.1 to 0.3) |
|  |  | follow up 24 weeks | - | distract/ignore: d=0.1(95% CI -0.1 o 0.3); Catastrophizing: d=-0.1(95% CI -0.3 to 0.1), |  | distract/ignore: d=0.0(95% CI -0.2 to 0.3); Catastrophizing: d=-0.0(95% CI -0.2 to 0.1) |
|  |  | follow up 48 weeks | - | distract/ignore: d=0.1(95% CI -0.1 to 0.4); Catastrophizing: d=-0.3(95% CI -0.5 to -0.1); | - | distract/ignore: d=-0.0(95% CI -0.3 to 0.2); Catastrophizing: d=0.0(95% CI -0.2 to 0.2); |
|  | Pain beliefs and coping (Parent Report). PBQ | follow up 1 week | - | threat of child pain: d=-0.2(95% CI -0.3 to 0.1); emotion-focused: d=0.3(95% CI 0.1 to 0.4); problem-focused: d=0.5(95% CI 0.3 to 0.7) | - | threat of child pain: d=-0.0(95% CI -0.1 to 0.1); emotion-focused: d=0.2(95% CI 0.1 to 0.3); problem-focused: d=0.0(95% CI -0.1 to 0.2) |
|  |  | follow up 12 weeks | - | threat of child pain: d=-0.1(95% CI -0.3 to 0.0); emotion-focused: d=0.3(95% CI 0.1 to 0.4); problem-focused: d=0.5(95% CI 0.3 to 0.6) | - | threat of child pain: d=-0.1(95% CI -0.3 to 0.0); emotion-focused: d=0.2(95% CI 0.1 to 0.3); problem-focused: d=0.2(95% CI -0.0 to 0.3) |
|  |  | follow up 24 weeks | - | threat of child pain: d=-0.2(95% CI -0.3-0.0); emotion-focused: d=0.3(95% CI 0.2,0.4); problem-focused: d=0.4(95% CI 0.2,0.5) | - | threat of child pain: d=-0.1(95% CI -0.3,-0.0); emotion-focused: d=0.1(95% CI 0.0,0.3); problem-focused: d=0.1(95% CI -0.1,0.2) |
|  |  | follow up 48 weeks | - | threat of child pain: d=-0.2(95% CI -0.4-0.1); emotion-focused: d=0.4(95% CI 0.2,0.5); problem-focused: d=0.4(95% CI 0.2,0.5) | - | threat of child pain: d=-0.1(95% CI -0.3,0.0); emotion-focused: d=0.2(95% CI 0.1,0.4); problem-focused: d=0.0(95% CI -0.2,0.2); |
|  | Pain beliefs and coping (Child Report). PBQ | follow up 1 week | - | child perceived pain: d=-0.1(95% CI -0.2,0.1); emotion-focused: d=0.2(95% CI 0.1,0.4); problem-focused: d=0.4(95% CI 0.2,0.5) | - | child perceived pain: d=0.1(95% CI -0.0,0.2); emotion-focused: d=0.1(95% CI -0.1,0.3); problem-focused: d=0.3(95% CI 0.1,0.4) |
|  |  | follow up 12 weeks | - | child perceived pain: d=-0.1(95% CI -0.2,0.1); emotion-focused: d=0.2(95% CI 0.1,0.4); problem-focused: d=0.4(95% CI 0.2,0.5) | - | child perceived pain: d=0.0(95% CI -0.2,0.2); emotion-focused: d=0.1(95% CI -0.1,0.3); problem-focused: d=0.2(95% CI -0.0,0.4) |
|  |  | follow up 24 weeks | - | child perceived pain: d=-0.095% CI (-0.2,0.1); emotion-focused: d=0.1(95% CI -0.0,0.3); problem-focused: d=0.2(95% CI -0.0,0.4) | - | child perceived pain: d=0.0(95% CI -0.2,0.2); emotion-focused: d=0.1(95% CI 0.0,0.3); problem-focused: d=-0.1(95% CI -0.3,0.1) |
|  |  | follow up 48 weeks | - | Child report child perceived pain: d=-0.1(95% CI -0.2,0.1); emotion-focused: d=0.2(-0.0,0.3); problem-focused: d=0.1(95% CI -0.1,0.4) | - | child perceived pain: d=0.1(95% CI -0.1,0.2); emotion-focused: d=0.2(95% CI 0.0,0.3); problem-focused: d=-0.1(95% CI -0.3,0.2) |
| Thompson, R 2012 | Psychiatric Diagnoses, K-SADS-PL* | post intervention (12W) | 21 | 0.67 (95% CI 0.33, 1.02) | 19 | 1.32 (95% CI 0.96, 1.68) |
|  |  | follow up 36 weeks | 19 | 0.45 (0.06, 0.84) | 16 | 0.72 (95% CI 0.30, 1.14) |
|  |  | follow up 12 weeks | 18 | 0.53 (95% CI 0.16, 0.89) | 15 | 0.63 (95% CI 0.23, 1.02) |
|  | Global Functioning, CGAS* | post-intervention (12W) | 21 | 69.5 (95% CI 66.1, 72.9) | 19 | 63.0 (95% CI 59.4, 66.6) |
|  |  | follow up 36 weeks | 19 | 70.1 (95% CI 67.1, 73.1) | 16 | 64.8 (95% CI 61.5, 68.1) |
|  |  | follow up 12 weeks | 18 | 69.8 (95% CI 66.2, 73.4) | 15 | 66.7 (95% CI 62.8, 70.6) |

SFQ - Social Functioning Questionnaire; COPE - Brief Coping Operations Preference Enquiry; IBDSCCQ - IBD Stages of Change Coping Questionnaire; TTM - Trans-Theoretical Model of behavioural change; HB - Hemoglobin; WCC - White cell count; K-SADS-PL - Schedule for Affective Disorders and Schizophrenia for School-Age Children-Present and Lifetime Version; PCSC - Perceived Control Scale for Children; CGAS - Children`s Global Assessment Scale; FFMQ - Five Facet Mindfulness Questionnaire ;VSI - Visceral Sensitivity Index; GI-COG - Gastrointestinal Cognitions Questionnaire; ARCS - Adults’ Responses to Children’s Symptoms; PRI - Pain Response Inventory; PBQ - Pain Beliefs Questionnaire;

*ITT data

### Additional file 1: Table S4 Excluded studies

|  | Year | Title | DOI | Reason for exclusion |
| --- | --- | --- | --- | --- |
| 1 | 2011 | INSPIRE study: Does stress management improve the course of inflammatory bowel disease and disease-specific quality of life in distressed patients with ulcerative colitis or crohn's disease? A randomized controlled trial | 10.1002/ibd.21575 | Inappropriate intervention |
| 2 | 2004 | Social learning, affective state and passive coping in irritable bowel syndrome and inflammatory bowel disease | 10.1016/j.genhosppsych.2003.07.005 | Inappropriate intervention |
| 3 | 2005 | Effects of mind-body therapy on quality of life and neuroendocrine and cellular immune functions in patients with ulcerative colitis | 10.1159/000086318 | Inappropriate intervention |
| 4 | 2011 | Behavioral interventions may prolong remission in patients with inflammatory bowel disease | 10.1016/j.brat.2010.12.005 | Inappropriate intervention |
| 5 | 2004 | Effect of psychotherapy and relaxation on the psychosocial and somatic course of Crohn's disease - Main results of the German Prospective Multicenter Psychotherapy Treatment Study on Crohn's Disease | 10.1016/s0022-3999(03)00122-3 | Inappropriate intervention |
| 6 | 2015 | Genomic and clinical effects associated with a relaxation response mind-body intervention in patients with irritable bowel syndrome and inflammatory bowel disease | 10.1371/journal.pone.0123861 | Inappropriate intervention |
| 7 | 2018 | A Multidisciplinary Approach to Biopsychosocial Care for Adults With Inflammatory Bowel Disease: A Pilot Study | 10.1093/ibd/izy215 | Inappropriate intervention |
| 8 | 2018 | Cognitive behavioural therapy for the management of inflammatory bowel diseasefatigue: A pilot randomised controlled trial | 10.1093/ecco-jcc/jjx180.1012 | Conferences paper |
| 9 | 2016 | The beneficial effects of mindfulness training on fatigue and psychosocial functioning in patients with inflammatory bowel disease and irritable bowel syndrome: A pilot study | 10.1093/ecco-jcc/jjw019 | Conferences paper |
| 10 | 2012 | The effectiveness of cognitive behavioral therapy on the quality of life of patients with inflammatory bowel disease: multi-center design and study protocol (KL!C- study) | [10.1186/1471-244x-12-227](http://www.sciencedirect.com/science/article/pii/S000579671400014X) | Trials protocol |
| 11 | 2019 | Mindfulness-Based Cognitive Therapy Experiences in Youth With Inflammatory Bowel Disease and Depression: Protocol for a Mixed Methods Qualitative Study | 10.2196/14432 | Trials protocol |
| 12 | 2016 | Effectiveness of disease-specific cognitive-behavioural therapy on depression, anxiety, quality of life and the clinical course of disease in adolescents with inflammatory bowel disease: study protocol of a multicentre randomised controlled trial (HAPPY-IBD) | 10.1136/bmjgast-2015-000071 | Trials protocol |
| 13 | 2019 | Feasibility and acceptability of a mindfulness-based group intervention for adolescents with inflammatory bowel disease | 10.1007/s10880-019-09622-6 | Inappropriate study design, there was no control group |
| 14 | 2018 | Low-intensity, evidence-based cognitive-behavioural therapy of a patient with Crohn's disease | 10.1556/650.2018.30969 | Inappropriate study design, there was no control group |
| 15 | 2014 | A randomized controlled trial of mindfulness-based stress reduction to prevent flare-up in patients with inactive ulcerative colitis | 10.1159/000356316 | Inappropriate method in the control group, the control group also used psychotherapy |
| 16 | 2009 | Psychiatric liaison nursing intervention method for stress management: relaxation and cognitive behavioral therapy in inflammatory bowel disease patients | 00258512 (ISSN) | Inappropriate study design, there was no control group |
| 17 | 2003 | Reducing psychological distress in patients with inflammatory bowel disease by cognitive-behavioural treatment: exploratory study of effectiveness | 10.1080/00365520310003110 | Inappropriate study design, there was no control group |
| 18 | 2013 | Integrating illness concerns into cognitive behavioral therapy for children and adolescents with inflammatory bowel disease and co-occurring anxiety | 10.1111/jspn.12019 | Inappropriate study design, there was no control group |
| 19 | 2003 | Evidenced-based psychotherapy with chronic inflammatory bowel disease patients |  | Review |
| 20 | 2017 | Psychological Interventions for Irritable Bowel Syndrome and Inflammatory Bowel Diseases | 10.1038/ctg.2016.69 | Review |
| 21 | 2007 | Is there a benefit from intensified medical and psychological interventions in patients with functional dyspepsia not responding to conventional therapy? | 10.1111/j.1365-2036.2007.03277.x | Not relevant topic, this article is not about IBD |
| 22 | 2017 | Self-administered Cognitive Behavior Therapy for IBS: The IBS Outcome Study (IBSOS) Randomized Clinical Trial | 10.14309/00000434-201710001-00422 | Not relevant topic, this article is not about IBD |
| 23 | 2006 | Effectiveness of a cognitive-behavioural program to reduce anxiety in medically ill patients |  | Not relevant topic, this article is not about IBD |
| 24 | 2015 | The Effectiveness of Acceptance and Commitment Therapy on Stress Coping Strategies in Women with Ulcerative Colitis |  | Incomplete data |
| 25 | 2015 | Treatment for comorbid pediatric gastrointestinal and anxiety disorders: A pilot study of a flexible health sensitive cognitive-behavioral therapy program | 10.1037/cpp0000116 | Inappropriate method in the control group, the control group also used psychotherapy |
| 26 | 2018 | اثربخشی مداخله ی مبتنی بر ذهن آگاهی بر فعالیت بیماری و کیفیت زندگی بیماران مبتلا به کولیت اولسراتیو |  | Incomplete data |
| 27 | 2007 | Tratamiento cognitivo-conductual protocolizado en grupo de las enfermedades inflamatorias intestinales | 10.4321/s1130-01082007001000006 | Incomplete data |
| 28 | 2014 | Randomized efficacy trial of two psychotherapies for depression in youth with inflammatory bowel disease | 10.1016/j.jaac.2014.04.014 | Inappropriate method in the control group, the control group also used psychotherapy |
| 29 | 2015 | Effect of 2 psychotherapies on depression and disease activity in pediatric Crohn's disease | 10.1097/MIB.0000000000000358 | Inappropriate method in the control group, the control group also used psychotherapy |
| 30 | 2019 | Effect of Cognitive Behavioral Therapy on Clinical Disease Course in Adolescents and Young Adults With Inflammatory Bowel Disease and Subclinical Anxiety and/or Depression: results of a Randomized Trial | 10.1093/ibd/izz073 | Incomplete data |
| 31 | 2007 | Protocolized cognitive-behavioural group therapy for inflammatory bowel disease | - 18052663 (PMID) | Data overlapping with another  Paper |
| 32 | 2015 | A Controlled Study of a Group Mindfulness Intervention for Individuals Living Wiith Inflammatory Bowel Disease | [10.1097/MIB.0000000000000629](http://dx.doi.org/10.1097/MIB.0000000000000629) | Inappropriate study design, it was a non-randomized controlled trial |

Additional file 1: Appendix S1 Searching queries in PubMed

| Recent queries in PubMed | |  |
| --- | --- | --- |
| Search | Query | Items found |
| #1 | Search (((Inflammatory bowel diseases[MeSH Terms]) OR Inflammatory Bowel Disease) OR Bowel Diseases, Inflammatory) OR IBD | 103377 |
| #2 | Search ((((((((((((((((Crohn Disease[MeSH Terms]) OR Crohn's Enteritis) OR Regional Enteritis) OR Crohn's Disease) OR Crohn's disease) OR Inflammatory Bowel Disease 1) OR Enteritis, Granulomatous) OR Granulomatous Enteritis) OR Enteritis, Regional) OR Ileocolitis) OR Colitis, Granulomatous) OR Granulomatous Colitis) OR Ileitis, Terminal) OR Terminal Ileitis) OR Ileitis, Regional) OR Regional Ileitides) OR Regional Ileitis | 55952 |
| #3 | Search ((((Colitis, Ulcerative[MeSH Terms]) OR Idiopathic Proctocolitis) OR Ulcerative Colitis) OR Colitis Gravis) OR Inflammatory Bowel Disease, Ulcerative Colitis Type | 47215 |
| #4 | Search (Proctocolitis[MeSH Terms]) OR (Rectocolitis OR Rectocolitis, Hemorrhagic OR Hemorrhagic Rectocolitis OR Proctocolitis, Hemorrhagic OR Hemorrhagic Proctocolitis OR Rectocolitis, Ulcerative OR Rectocolitides, Ulcerative OR Ulcerative Rectocolitides OR Ulcerative Rectocolitis OR Ulcerative Proctocolitis OR Proctocolitis, Ulcerative OR Proctocolitides, Ulcerative OR Ulcerative Proctocolitides OR Proctosigmoiditis OR Rectosigmoiditis OR) | 47964 |
| #5 | Search (proctitis[MeSH Terms]) OR Proctitides | 4316 |
| #6 | Search pancolitis | 701 |
| #7 | Search (Enterocolitis[MeSH Terms]) OR Enterocolitides | 18192 |
| #8 | #1 OR #2 OR #3 OR #4 OR #5 OR #6 OR #7 | 134588 |
| #9 | Search ((((Behavioral Therapies, Cognitive Behavioral Therapy, Cognitive Cognitive Behavioral Therapies Therapies, Cognitive Behavioral Therapy, Cognitive Behavioral Therapy, Cognition Therapy, Cognitive Behavior Cognition Therapy OR Cognition Therapies OR Therapies, Cognition OR Cognitive Psychotherapy OR Cognitive Psychotherapies OR Psychotherapies, Cognitive OR Psychotherapy, Cognitive OR Therapy, Cognitive OR Cognitive Therapies OR Therapies, Cognitive OR Cognitive Therapy OR Cognitive Behavior Therapy OR Behavior Therapies, Cognitive OR Cognitive Behavior Therapies OR Therapies, Cognitive Behavior OR Behavior Therapy, Cognitive)) OR Cognitive Behavioral Therapy[MeSH Terms])) OR CBT | 106617 |
| #10 | Search (Acceptance and Commitment Therapy[MeSH Terms]) | 327 |
| #11 | Search Mindfulness[MeSH Terms] | 2778 |
| #12 | Search (Behavior Therapy[MeSH Terms]) OR (Conditioning Therapy OR Therapy, Conditioning OR Conditioning Therapies OR Therapies, Conditioning OR Behavior Modification OR Behavior Modifications OR Modification, Behavior OR Modifications, Behavior OR Therapy, Behavior OR Behavior Therapies OR Therapies, Behavior) | 235075 |
| #13 | #10 OR #11 OR #12 | 301023 |
| #14 | Search (((((((((((randomized controlled trial[Publication Type]) OR controlled clinical trial[Publication Type]) OR randomized[Title/Abstract]) OR placebo[Title/Abstract]) OR drug therapy[MeSH Subheading]) OR randomly[Title/Abstract]) OR trial[Title/Abstract]) OR groups[Title/Abstract])) NOT (((animals[MeSH Terms]) NOT humans[MeSH Terms])))) | 4027365 |
| #15 | #8 AND #13 AND #14 | 419 |

2019/12/8 P.M 2:39:11

Additional file 1: Appendix S2 Searching queries in Embase

Embase 2019/12/8

| No. | Query | Results |
| --- | --- | --- |
| #1 | 'psychotherapy'/exp | 263799 |
| #2 | 'behavior therapy'/exp | 45141 |
| #3 | cbt:ti,ab,kw | 15762 |
| #4 | cognitiv* NEAR/3 (behav* OR treatment* OR technique* OR therap* OR intervention* OR restructur* OR reappraisal*) | 124938 |
| #5 | behav* NEAR/3 (treatment* OR therap* OR intervention* OR activat* OR technique* OR modif* OR change*) | 242090 |
| #6 | coping* NEAR/3 (skill* OR strateg*) | 23235 |
| #7 | psychotherap* | 181238 |
| #8 | psychological* | 866687 |
| #9 | talk* NEAR/3 (therap* OR intervention*) | 930 |
| #10 | mindful* | 13836 |
| #11 | acceptance* NEAR/2 commitment* | 1684 |
| #12 | 'crossover procedure':de OR 'double-blind procedure':de OR 'randomized controlled trial':de OR 'single-blind procedure':de OR random*:de,ab,ti OR factorial*:de,ab,ti OR crossover*:de,ab,ti OR ((cross NEXT/1 over*):de,ab,ti) OR placebo*:de,ab,ti OR ((doubl* NEAR/1 blind*):de,ab,ti) OR ((singl* NEAR/1 blind*):de,ab,ti) OR assign*:de,ab,ti OR allocat*:de,ab,ti OR volunteer*:de,ab,ti | 2502527 |
| #13 | 'inflammatory bowel disease'/exp | 150671 |
| #14 | inflammatory AND bowel AND disease* | 97203 |
| #15 | **ibd** | 54463 |
| #16 | crohn* | 107842 |
| #17 | ulcerat* AND colitis | 84472 |
| #18 | pancolitis | 2375 |
| #19 | proctitis | 7339 |
| #20 | proctocolitis | 1187 |
| #21 | 'enterocolitis'/exp | 5662 |
| #22 | 'ulcerative colitis'/exp | 75170 |
| #23 | 'crohn disease'/exp | 90607 |
| #24 | #13 OR #14 OR #15 OR #16 OR #17 OR #18 OR #19 OR #20 OR #21 OR #22 OR #23 | 204774 |
| #25 | #1 OR #2 OR #3 OR #4 OR #5 OR #6 OR #7 OR #8 OR #9 OR #10 OR #11 | 1314489 |
| #26 | #12 AND #24 AND #25 | 553 |

窗体底端

Search Name: cochrane

Date Run: 08/12/2019 17:06:08

Comment: CBT and IBD

| ID | Search | Hits |
| --- | --- | --- |
| #1 | MeSH descriptor: [Inflammatory Bowel Diseases] explode all trees | 2987 |
| #2 | crohn* or IBD or (inflammatory bowel disease*) or (ulcerative colitis) or colitis | 14130 |
| #3 | MeSH descriptor: [Behavior Therapy] explode all trees | 15593 |
| #4 | CBT:TI,AB,KW | 7224 |
| #5 | cognitiv* NEAR3 (behav* or treatment* or technique* or therap* or intervention* or restructur* or reappraisal*) | 486 |
| #6 | behav* NEAR3 (treatment* OR therap* or intervention* OR activat* or technique* or modif* or change*) | 531 |
| #7 | coping* NEAR3 (skill* or strateg*) | 74 |
| #8 | talk* NEAR3 (therap* or intervention*) | 360 |
| #9 | mindful* | 4719 |
| #10 | acceptance* NEAR commitment* | 939 |
| #11 | MeSH descriptor: [Enterocolitis] explode all trees | 396 |
| #12 | pancolitis | 96 |
| #13 | MeSH descriptor: [Proctitis] explode all trees | 132 |
| #14 | MeSH descriptor: [Proctocolitis] explode all trees | 24 |
| #15 | #1 or #2 or #11 or #12 or #13 or #14 | 14484 |
| #16 | #3 or #4 or #5 or #6 or #7 or #8 or #9 or #10 | 24560 |
| #17 | #15 and #16 | 233 |

Additional file 1: Appendix S3 Searching queries in CINAHL with Full Text

EBSCOhost Research Databases - CINAHL with Full Text

Sunday, December 08, 2019 4,:29:37 AM

| **#** | **Query** | **Results** |
| --- | --- | --- |
| **S4** | **S1 AND S2 AND S3** | **112** |
| S3 | ( (MH "Random Assignment") or (MH "Random Sample+") or (MH "Crossover Design") or (MH "Clinical Trials+") or (MH "Comparative Studies") or (MH "Control (Research)+") or (MH "Control Group") or (MH "Factorial Design") or (MH "Quasi-Experimental Studies+") or (MH "Placebos") or (MH "Meta Analysis") or (MH "Sample Size") or (MH "Research, Nursing") or (MH "Research Question") or (MH "Research Methodology+") or (MH "Evaluation Research+") or (MH "Concurrent Prospective Studies") or (MH "Prospective Studies") or (MH "Nursing Practice, Research-Based") or (MH "Solomon Four-Group Design") or (MH "One-Shot Case Study") or (MH "Pretest-Posttest Design+") or (MH "Static Group Comparison") or (MH "Study Design") or (MH "Clinical Research+") ) or ( clinical nursing research or random* or cross?over or placebo* or control* or factorial or sham* or meta?analy* or systematic review* or blind* or mask* or trial* ) | 2,640,636 |
| S2 | TI ( behavior therap* or behavior modification* or behavior intervention* ) OR AB ( behavior therap* or behavior modification* or behavior intervention* ) OR TI ( cognitive behavioral therap* or cbt or cognitive behavioural therap* or cognitive behavior therap* ) OR AB ( cognitive behavioral therap* or cbt or cognitive behavioural therap* or cognitive behavior therap* ) OR TI ( mindful* or meditation* ) OR AB ( mindful* or meditation* ) OR TI ( acceptance and commitment therap* or act or mindfulness based therap* ) OR AB ( acceptance and commitment therap* or act or mindfulness based therap* ) | 80,200 |
| S1 | TI ( (Inflammatory bowel OR IBD OR Crohn* OR ulcerative colitis OR enterocolitis OR pancolitis OR proctitis OR proctocolitis) ) OR AB ( (Inflammatory bowel OR IBD OR Crohn* OR ulcerative colitis OR enterocolitis OR pancolitis OR proctitis OR proctocolitis) ) | 16,892 |

Additional file 1: Appendix S4 Searching queries in PsycINFO

Sunday, December 08, 2019 ,4:39:45 AM

EBSCOhost Research Databases
PsycINFO

| **#** | **Query** | **Result** |
| --- | --- | --- |
| **S4** | S1 AND S2 AND S3 | **37** |
| S3 | ( (MH "Random Assignment") or (MH "Random Sample+") or (MH "Crossover Design") or (MH "Clinical Trials+") or (MH "Comparative Studies") or (MH "Control (Research)+") or (MH "Control Group") or (MH "Factorial Design") or (MH "Quasi-Experimental Studies+") or (MH "Placebos") or (MH "Meta Analysis") or (MH "Sample Size") or (MH "Research, Nursing") or (MH "Research Question") or (MH "Research Methodology+") or (MH "Evaluation Research+") or (MH "Concurrent Prospective Studies") or (MH "Prospective Studies") or (MH "Nursing Practice, Research-Based") or (MH "Solomon Four-Group Design") or (MH "One-Shot Case Study") or (MH "Pretest-Posttest Design+") or (MH "Static Group Comparison") or (MH "Study Design") or (MH "Clinical Research+") ) or ( clinical nursing research or random* or cross?over or placebo* or control* or factorial or sham* or meta?analy* or systematic review* or blind* or mask* or trial* ) | 1,030,664 |
| S2 | TI ( (Inflammatory bowel OR IBD OR Crohn* OR ulcerative colitis OR enterocolitis OR pancolitis OR proctitis OR proctocolitis) ) OR AB ( (Inflammatory bowel OR IBD OR Crohn* OR ulcerative colitis OR enterocolitis OR pancolitis OR proctitis OR proctocolitis) ) | 1,743 |
| S1 | TI ( behavior therap* or behavior modification* or behavior intervention* ) OR AB ( behavior therap* or behavior modification* or behavior intervention* ) OR TI ( cognitive behavioral therap* or cbt or cognitive behavioural therap* or cognitive behavior therap* ) OR AB ( cognitive behavioral therap* or cbt or cognitive behavioural therap* or cognitive behavior therap* ) OR TI ( mindful* or meditation* ) OR AB ( mindful* or meditation* ) OR TI ( acceptance and commitment therap* or act or mindfulness based therap* ) OR AB ( acceptance and commitment therap* or act or mindfulness based therap* ) | 176,622 |

Additional file 1: Appendix S5 Searching queries in Web of Science

| Databases= WOS, BCI, KJD, MEDLINE, RSCI, SCIELO Timespan=All years  Search language=Auto | |  |
| --- | --- | --- |
| Set | Query | Results |
| #1 | TOPIC: (Crohn*) | 99,365 |
| #2 | TOPIC: (Ulcerative colitis) | 82,411 |
| #3 | TOPIC: (IBD) | 43,235 |
| #4 | TOPIC: (Inflammatory bowel disease*) | 114,213 |
| #5 | TOPIC: (enterocolitis) | 25,136 |
| #6 | TOPIC: (pancolitis) | 1,187 |
| #7 | TOPIC: (proctitis) | 5,080 |
| #8 | TOPIC: (proctocolitis) | 1,329 |
| #9 | #8 OR #7 OR #6 OR #5 OR #4 OR #3 OR #2 OR #1 | 225,237 |
| #10 | TOPIC: (Cognitive Behavioral Therap*) | 181,664 |
| #11 | TOPIC: (Cognition Therap*) | 132,654 |
| #12 | TOPIC: (Cognitive Psychotherap*) | 20,508 |
| #13 | TOPIC: (Cognitive Therap*) | 260,662 |
| #14 | TOPIC: (Cognitive Behavior Therap*) | 172,665 |
| #15 | TOPIC: (Acceptance and Commitment Therapy) | 2,892 |
| #16 | TOPIC: (Mindfulness) | 16,435 |
| #17 | TOPIC: (Psychotherap*) | 122,705 |
| #18 | #17 OR #16 OR #15 OR #14 OR #13 OR #12 OR #11 OR #10 | 409,104 |
| #19 | TS= clinical trial* OR TS=research design OR TS=comparative stud* OR TS=evaluation stud* OR TS=controlled trial* OR TS=follow-up stud* OR TS=prospective stud* OR TS=random* OR TS=placebo* OR TS=(single blind*) OR TS=(double blind*) | 10,036,452 |
| #20 | #19 AND #18 AND #9 | 444 |

2019/12/08

Additional file 1: Appendix S6 Searching queries in Clinical trials

**. Gov 2019/12/8/19:26**

**CBT OR Cognitive Behavioral Therapy OR Acceptance and Commitment Therapy OR Mindfulness OR Behavior Therapy | Inflammatory Bowel Diseases OR Ulcerative colitis OR Crohn’s Disease 72**
